# Iron Bioavailability and Provitamin A from Sweet Potato- and Cereal-Based Complementary Foods

**DOI:** 10.3390/foods4030463

**Published:** 2015-09-18

**Authors:** Tatiana Christides, Francis Kweku Amagloh, Jane Coad

**Affiliations:** 1 Department of Life & Sports Sciences, Faculty of Engineering & Science, University of Greenwich, Medway Campus, Chatham Maritime, Kent ME4 4TB, UK; 2 Food Processing Technology Unit, Faculty of Agriculture, University for Development Studies, Ghana; E-Mail: fkamagloh@uds.edu.gh; 3 School of Food and Nutrition, Massey Institute of Food Science and Technology, College of Health, Te Kura Hauora Tangata, Massey University, Private Bag 11222, Palmerston North 4442, New Zealand; E-Mail: j.coad@massey.ac.nz

**Keywords:** bioavailability, Caco-2 cell, complementary food, β-carotene, iron, sweet potato, polyphenols, vitamin A

## Abstract

Iron and vitamin A deficiencies in childhood are public health problems in the developing world. Introduction of cereal-based complementary foods, that are often poor sources of both vitamin A and bioavailable iron, increases the risk of deficiency in young children. Alternative foods with higher levels of vitamin A and bioavailable iron could help alleviate these micronutrient deficiencies. The objective of this study was to compare iron bioavailability of β-carotene-rich sweet potato-based complementary foods (orange-flesh based sweet potato (OFSP) ComFa and cream-flesh sweet potato based (CFSP) ComFa with a household cereal-based complementary food (Weanimix) and a commercial cereal (Cerelac^®^), using the *in vitro* digestion/Caco-2 cell model. Iron bioavailability relative to total iron, concentrations of iron-uptake inhibitors (fibre, phytates, and polyphenols), and enhancers (ascorbic acid, ß-carotene and fructose) was also evaluated. All foods contained similar amounts of iron, but bioavailability varied: Cerelac^®^ had the highest, followed by OFSP ComFa and Weanimix, which had equivalent bioavailable iron; CFSP ComFa had the lowest bioavailability. The high iron bioavailability from Cerelac^®^ was associated with the highest levels of ascorbic acid, and the lowest levels of inhibitors; polyphenols appeared to limit sweet potato-based food iron bioavailability. Taken together, the results do not support that CFSP- and OFSP ComFa are better sources of bioavailable iron compared with non-commercial/household cereal-based weaning foods; however, they may be a good source of provitamin A in the form of β-carotene.

## 1. Introduction

Iron deficiency (ID) and Vitamin A deficiency (VAD) are prevalent in the developing world, especially in vulnerable groups such as infants and young children [1]. These micronutrient deficiencies are particularly problematic in young children; both ID and VAD during infancy can cause long-term health problems that cannot be reversed even with adequate intake later in life [2,3]. Infants and toddlers are at increased risk for ID and VAD when complementary foods, which are usually cereal-based, are introduced [4]. These cereal-based foods are often poor dietary sources of both vitamin A [5,6] and bioavailable iron as they contain high levels of iron absorption inhibitors such as phytates [7,8], or polyphenols [8]. The ideal complementary food should have adequate levels of nutrients and high mineral bioavailability [9]. VAD and ID often co-exist in low-income countries due to long-term poverty and plant-based monotonous diets [1], thus sustainable strategies, particularly food-based approaches, which will lead to high intake of dietary vitamin A and bioavailable iron are desirable.

Sweet potato-based complementary food formulations, prepared from cream- or orange-fleshed sweet potato (CFSP or OFSP, respectively) cultivars, contain significantly more β-carotene, the precursor of vitamin A, than cereal-based products [5,6]; have acceptable sensory attributes [10,11]; and, desirable physical properties, such as viscosity, for complementary feeding [10,11]. Additionally, sweet potato is relatively low in phytate compared to cereals [12,13], thus it would be expected that the sweet potato food matrix would have a less inhibitory effect on iron absorption in human infants. Furthermore, compositional analysis of sweet potato-based complementary foods found high concentrations of ascorbic acid and fructose [6]; ascorbic acid is a known enhancer of iron uptake [14], and research indicates that both ß-carotene and fructose may also improve iron bioavailability [15,16]. Therefore, it would be predicted that sweet potato-based complementary foods, with comparable amounts of iron as in cereal-based infant foods, would have better iron bioavailability. There is support for this in the research literature. In an *in vitro* study conducted by Lung’aho & Glahn (2009), the trend of data suggested that the addition of sweet potato (cultivar not specified) to food mixes potentiated iron bioavailability from household-level recipes [17], and a review suggested that sweet potato, particularly the OFSP food matrix, would be expected to promote iron absorption [18].

Sweet potato, however, is also a rich source of polyphenols [19,20]. Polyphenols are potent inhibitors of non-haem iron (inorganic iron and the most prevalent form in the diet) uptake, and may even inhibit absorption of haem-iron [21,22,23]. Studies have demonstrated that tannic acid (a commonly occurring dietary polyphenol) at a 0.1:1 tannin to iron molar ratio decreased iron uptake by almost 100% in the Caco-2 cell model [24], and a single meal study found that a tannic acid:iron 1:1 molar ratio led to almost 90% decreased iron absorption [25]. In addition, different sweet potato cultivars may contain different types of polyphenols [19,20]; the iron inhibitory effects of polyphenols vary with structure, thus knowing only total levels of food polyphenols may limit the ability to predict their effects on iron absorption [25,26,27].

Dietary iron bioavailability (outside of host factors) represents the sum of interactions between total iron levels, and iron uptake inhibitors and enhancers; proximate analysis can be used to predict iron absorption, but experiments directly measuring iron uptake are needed to more fully evaluate the net effects of complex food matrices on iron bioavailability.

The *in vitro* digestion/Caco-2 cell model, using cell ferritin formation as a surrogate marker for iron uptake, is a validated method for initial screening of food iron availability [28]. Furthermore, it has specifically been applied to evaluate iron bioavailability from complementary foods [17,29]. Therefore, this model can be used to assess iron bioavailability of sweet potato-based household-level food formulations as alternatives to cereal-based complementary foods.

The objective of this study was to assess the iron bioavailability from sweet potato-based complementary food formulations (OFSP ComFa and CFSP ComFa) in comparison with a non-commercial cereal-based infant food composed of maize-soyabean-groundnut (Weanimix: a food blend developed by the Ghanaian Ministry of Health Nutrition Division and the United Nations Children’s Fund in 1987), and a commercial infant cereal (Cerelac^®^), using the Caco-2 cell *in vitro* digestion model. In addition, a further objective of this study was the evaluation of iron bioavailability in the tested food formulations, in relation to concentrations of iron absorption inhibitors (phytates, total polyphenols, and total dietary fibre), and enhancers (ascorbic acid, ß-carotene and fructose).

## 2. Experimental Section

### 2.1. Complementary Food Samples

The food samples, as previously described [6], were packaged in air- and light-safe containers, and couriered from Massey University, New Zealand (NZ) to the University of Greenwich, United Kingdom (UK), for testing in the *in vitro* digestion/Caco-2 cell model to assess their iron bioavailability. The samples were stored at 4 °C in air and light tight containers prior to the *in vitro* digestion for use in the Caco-2 cell studies. Samples were coded, and the investigator conducting the *in vitro* digestion/Caco-2 cell studies was blinded to the composition (*i.e*., which formulation) of tested samples.

Infant formulations were prepared from a blend of OFSP or CFSP, soyabean, fish powder and soyabean oil [6]. An audio recording for the preparation of ComFa, the sweet potato-based complementary food, is available online [30]. Weanimix is a blend of maize (usually the white cultivar), soyabean/cowpea, and groundnut; in this study, soyabean was used, and the blend was further enriched with fish powder [6]. Cerelac^®^ is a commercial infant cereal commonly used in Africa; the sample evaluated in this study was Cerelac^®^ Infant Cereal Wheat & Ikan Bilis that also contained dried anchovies, and was donated by Nestlé Malaysia.

### 2.2. *In Vitro* Digestion/Caco-2 Model to Measure Iron Bioavailability

Two experiments were carried out. In Experiment I, iron bioavailability was investigated from all the formulations as prepared in NZ and received in the UK, herein referred to as “as provided”. In Experiment II, β-carotene alone, and β-carotene plus fructose, were used to fortify CFSP ComFa to levels equivalent with OFSP ComFa (13,353 μg β-carotene/100 g; and 13,353 μg β-carotene/100 g plus 7.15 g fructose/100 g, respectively) to investigate their effect on iron bioavailability. Analytical standard fructose and β-carotene were obtained from Sigma, solubilized, and added to the CFSP ComFa digest immediately prior to the beginning of the digestive process.

Iron uptake was assessed using the TC7 Caco-2 cell clone (INSERM U505) as used in previous studies [17,31].

In both experiments, 1 g of the food formulation was weighed, taking into account an adjustment for respective moisture content of different foods. The method used for the *in vitro* digestion procedure and iron bioavailability estimations has been published elsewhere [17,31].

Briefly, cells were grown in six-well tissue culture plates and maintained in DMEM supplemented with 10% *v*/*v* foetal bovine serum (FBS). On day 13, cell media was changed to MEM without FBS as described by Glahn *et al*. [28]. On day 14, foods were subjected to *in vitro* digestion with sequential addition of digestive enzymes to mimic exposure to the stomach and small intestine (pepsin at pH 2, followed by bile/pancreatin at pH 7). Digested foods (digestates), and controls including a blank “No food/added iron” digestate, were then applied to Caco-2 cells via an upper chamber suspended over the plate wells created using a 15-kD dialysis membrane fitted over a Transwell insert and held in place with a silicon ring (the membrane protects the cells from the digestive enzymes). Cells were treated for two hours, digestates removed, and the cells returned to the incubator. Twenty-four hours after the initiation of the digestive process cells were harvested for ferritin. Ferritin was measured using a commercial enzyme-linked immunosorbent assay (Spectro ferritin, RAMCO Laboratories Inc., Stafford, TX, USA), and corrected for differing cell numbers/wells by measurement of cell protein; cell protein was measured using the Pierce protein bicinchoninic acid assay (BCA). Ferritin values are expressed as ng ferritin/mg cell protein.

### 2.3. Compositional Analysis: β-Carotene, Vitamin C, Fructose, Iron, Total Polyphenols and Phytate

The methods used for determination of β-carotene, ascorbic acid (vitamin C), fructose, iron, total polyphenols and phytate have been described in depth in previous publications [6,32].

Briefly, levels of inhibitors were measured as follows. Phytates were measured using the K-PHYT 05/07 assay kit (Megazyme International, Bray, Co. Wicklow, Ireland); a quality control sample of oat flour was analysed alongside with samples and values were within the 10% variation as specified in the protocol. Sample polyphenol levels were measured using the redox Folin–Ciocalteu reagent, and quantified by UV/visible-light spectrophotometry; values are expressed as Gallic acid equivalents. Soluble and insoluble dietary fibres were determined by the enzymatic-gravimetric method (AOAC 991.43).

Levels of food components enhancing iron absorption were determined as follows. β-carotene was measured by the Carr–Price method (AOAC 974.29) using high-performance liquid chromatography (Shimadzu HPLC, Tokyo, Japan), ascorbic acid was measured by reversed-phase, high-performance liquid chromatography (HPLC), and fructose was determined by gas chromatography; all three assays were carried out by a commercial laboratory (Nutrition Laboratory, Massey University, New Zealand, accredited by the IANZ and a NZ Ministry of Health recommended agency).

Sample iron levels were measured by quadrupole inductively coupled mass spectrometry (Agilent 7500ce; Agilent Technologies Inc., Santa Clara, CA, USA) after high temperature digestion in 68% HNO3; analysis was conducted by the Campbell Microanalytical Laboratory, Department of Chemistry, University of Otago, New Zealand. Use of certified reference materials demonstrated a mean recovery of 103% for iron, which is within acceptable experimental standards.

### 2.4. Statistical Analysis

The data generated from the *in vitro* digestion/Caco-2 cell studies were analysed using general linear models. Data are presented as individual value plots. For the compositional data, One-Way ANOVA was used, and the results are presented as the mean ± SD. Tukey’s studentized range test was used to compare differences between means when the ANOVA result was significant (*p* < 0.05). Minitab 16.2.2 (Minitab Inc., State College, PA, USA) statistical package was employed for the statistical analysis.

## 3. Results

### 3.1. Iron Bioavailability of Tested Complementary Foods as Provided

**Figure 1:**
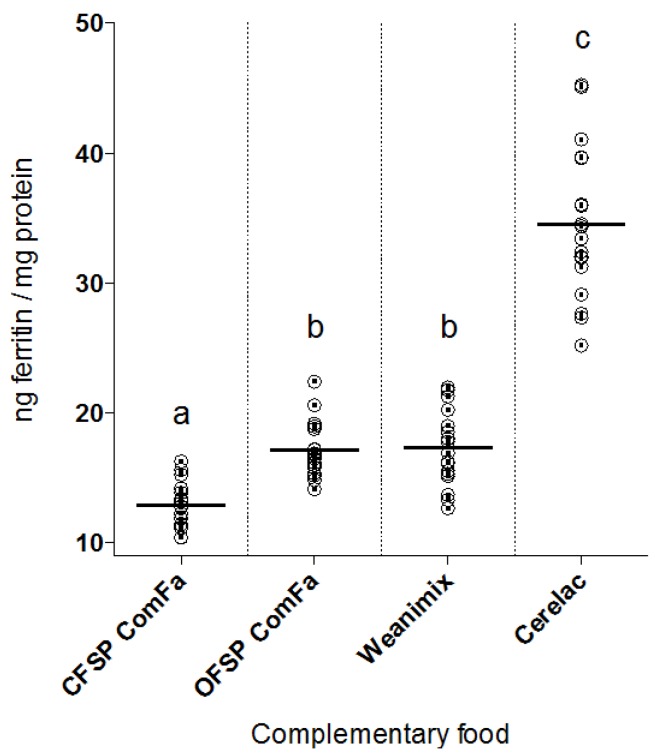
Ferritin formation per gram (adjusted for moisture content) of infant complementary weaning foods from: Cerelac^®^; CFSP ComFa; OFSP ComFa and Weanimix. Caco-2 cell ferritin formation was highest after exposure to the commercial infant cereal Cerelac^®^. OFSP ComFa and Weanimix ferritin levels were equivalent and approximately 50% less than Cerelac^®^ induced ferritin formation. CFSP ComFa treated cells formed the lowest amount of ferritin: 25% less than the levels formed in OFSP ComFa and Weanimix. Plots are individual values that have been normalised to the average blank digest (*i.e*., no added iron) ferritin level (*n* = 18); short horizontal lines indicate the group mean. Means per group with different letters are significantly different (*p* < 0.0001).

Data on iron bioavailability from the *in vitro* digestion/Caco-2 cell model for Experiment I are presented in Figure 1.

The commercial blend, Cerelac^®^, caused the greatest uptake of iron as measured by the ferritin assay; approximately 100% more than the average of that of the sweet potato- and maize-based blends investigated in this study. Iron uptake promoted by OFSP ComFa and Weanimix was equivalent, and approximately half that of Cerelac^®^ treated cells. CFSP ComFa treated cells had the lowest iron uptake; approximately 65% less than Cerelac^®^, and 25% less than both OFSP ComFa and Weanimix.

### 3.2. Iron Bioavailability of OFSP ComFa Compared with CFSP ComFa Fortified with Fructose and β-Carotene

**Figure 2:**
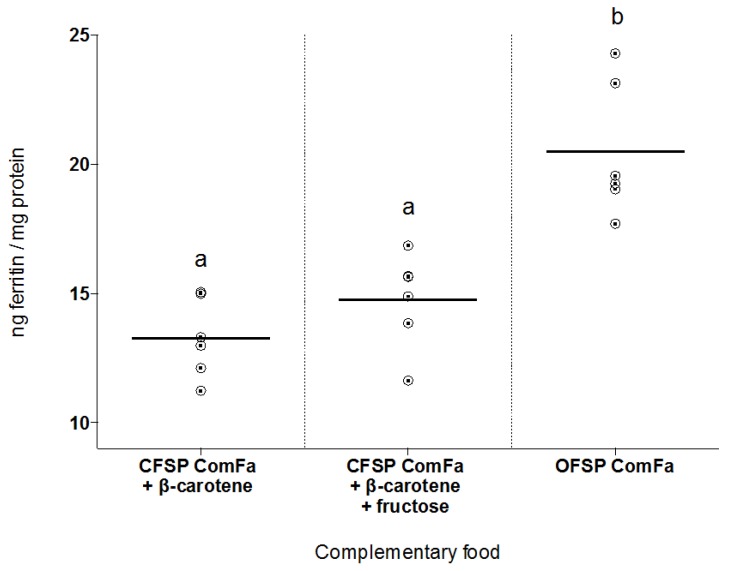
Ferritin formation per gram (adjusted for moisture content) of cream-fleshed sweet potato-based complementary food (CFSP ComFa) fortified with β-carotene, and β-carotene plus fructose, in comparison with orange-fleshed sweet potato-based food (OFSP ComFa). Ferritin formation was highest for Caco-2 cells exposed to OFSP ComFa; CFSP ComFa ferritin levels remained approximately 25% less than OFSP ComFa levels whether fortified with β-carotene alone, or β-carotene and fructose, and comparable to the CFSP ComFa ferritin concentration seen in Figure 1 (approximately 14 ng ferritin/mg protein). Plots are individual values that have been normalised to average blank ferritin levels (*n* = 6); short horizontal lines indicate the group mean. OFSP ComFa mean ferritin formation (labeled b) was significantly higher (*p* < 0.0001) than both other treatments (labeled a).

In Experiment I, with the complementary foods as provided, ferritin formation from OFSP ComFa was approximately 30% higher compared with CFSP ComFa. In Experiment II, the fortification of CFSP ComFa with the addition of β-carotene, and β-carotene and fructose, to levels equivalent to those in OFSP ComFa, did not increase ferritin levels in cells treated with fortified CFSP ComFa to the levels seen in cells treated with OFSP ComFa (Figure 2), and ferritin levels were comparable to those of unfortified CFSP ComFa (Figure 1). The difference in ferritin between fortified CFSP- and OFSP ComFa remained approximately 30%—*i.e.*, the same difference as between *unfortified* CFSP- and OFSP ComFa.

### 3.3. Concentrations of Total Iron and Iron Absorption Inhibitors in Tested Foods

Table 1 shows the data for total iron and iron absorption inhibitors.

All tested foods had similar levels of total iron. According to the manufacturer’s label, Cerelac^®^ was fortified with ferrous fumarate; data on the specific biochemical iron form of the other foods are not available.

Levels of inhibitors varied for the tested foods. Cerelac^®^ had the lowest levels of all measured inhibitors; however, this did not reach statistical significance for Cerelac^®^ polyphenol levels compared with Weanimix, nor Celerac^®^ phytate levels compared with CFSP ComFa. OFSP ComFa had the highest concentration of total dietary fibre (TDF), followed by CFSP ComFa (12.28 g/100 g and 10.05 g/100 g respectively). Weanimix TDF was approximately 50% of that in each of the sweet potato-based foods. Cerelac^®^ TDF was the lowest of all tested foods at 1.48 g/100 g.

Weanimix had the highest phytate levels: approximately twice that of OFSP ComFa, and approximately 5 times that of CFSP ComFa and Cerelac^®^, which had equivalent phytate concentrations.

The sweet potato-based foods had the highest levels of polyphenols, the most potent inhibitors of iron absorption; concentrations were nearly identical in OFSP- and CFSP ComFa, and approximately 80% and 100% higher than Weanimix and Cerelac^®^, respectively.

**Table 1:** Amount of inhibitors and enhancers of iron absorption, and the concentration of iron in sweet potato- and cereal-based complementary foods ^¶^.

Complementary Food	Inhibitor (/100 g)			Enhancer (/100 g)			Iron (mg/100 g)
	Phytate (mg)	Total Polyphenols (mg GA equ)	Total Dietary Fibre (g)	Ascorbic Acid (mg)	β-Carotene (µg)	Fructose (g)	
OFSP ComFa	229.85 ± 19.36 ^b^	466.28 ± 9.68 ^a^	12.28 ± 1.04 ^a^	32.48 ± 0.48 ^b^	13353 ± 1792.60 ^a^	7.15 ± 0.17 ^a^	7.76 ± 1.22 ^a^
CFSP ComFa	78.62 ± 3.50 ^c^	466.42 ± 34.98 ^a^	10.05 ± 0.18 ^b^	37.40 ± 0.61 ^a,b^	1263 ± 22.30 ^b^	3.09 ± 0.07 ^b^	7.26 ± 0.08 ^a^
Weanimix	438.10 ± 8.58 ^a^	263.68 ± 17.82 ^b^	6.90 ± 0.64 ^c^	BDL	34 ± 11.77 ^b^	BDL	6.53 ± 1.55 ^a^
Cerelac	66.92 ± 3.96 ^c^	213.45 ± 29.92 ^b^	1.48 ± 0.50 ^d^	53.11± 12.07 ^a^	66 ± 15.83 ^b^	BDL	8.85 ± 0.17 ^a^
*p-value*	*<0.0001*	*<0.0001*	*<0.0001*	*<0.0001*	*<0.0001*	*<0.0001*	*0.10*

^¶^ BDL, below detection limit. Values are the mean ± SD of triplicate determinations on dry matter basis; values within a column with unlike superscript letters are significantly different. Ascorbic acid, phytate, total polyphenols and fructose have previously been published [6]; Copyright 2014, The Nevin Scrimshaw International Nutrition Foundation (with permission).

### 3.4. Concentrations of Iron Absorption Enhancers in Tested Foods

Data on iron absorption enhancers are shown in Table 1.

Cerelac^®^ had the highest levels of ascorbic acid, an established enhancer of iron absorption [14]; levels were 50% higher than in either of the sweet potato-based foods. OFSP- and CFSP ComFa ascorbic acid levels were similar, and 500% higher than Weanimix, for which levels were below the detection limits (BDL) of the assay.

As predicted, OFSP ComFa had the highest concentration of β-carotene (13353 μg/100 g), followed by CFSP ComFa (1263 μg/100 g); levels were very low in Cerelac^®^ and Weanimix and not statistically different from one another.

Fructose concentrations were below the level of detection in both Cerelac^®^ and Weanimix. OFSP ComFa had the highest amount of fructose at 7.15 g/100 compared with 3.09 g/100 g in CFSP ComFa.

## 4. Discussion

As expected, Cerelac^®^ had the highest iron bioavailability as estimated by ferritin formation (Figure 1). Cerelac^®^ infant cereal is fortified with ferrous fumarate, the current recommended form of inorganic iron for fortification of complementary foods because of its relatively high bioavailability [33]. In contrast, the household formulations contained endogenous iron, which is typically in the less bioavailable ferric iron form [34]. Furthermore, levels of inhibitors were lower in Cerelac^®^; total dietary fibre (TDF) was statistically significant lower compared with all other tested food formulations. Although there is some debate about the role of TDF itself (versus the phytates it contains) in decreasing iron absorption in humans [35], the results of this study are consistent with other reports in the literature that would predict lower TDF would be associated with better iron bioavailability [36]. Concentrations of both polyphenols and phytates in Cerelac^®^ were also lower than the other formulations. In addition to lower concentrations of iron uptake inhibitors, Cerelac^®^ had approximately 50% higher levels of ascorbic acid. Cerelac^®^’s high iron bioavailability is thus explained by the combination of low levels of iron absorption inhibitors, high levels of ascorbic acid, and fortification with a relatively bioavailable form of inorganic iron.

Despite higher levels of iron absorption enhancers in OFSP ComFa compared with Weanimix, the results of this study indicate that the presence of inhibitors in the OF sweet potato-based formulation led to OFSP ComFa and Weanimix having similar levels of bioavailable iron as measured by ferritin formation (Figure 1)—approximately half the amount seen with Cerelac^®^. Proximate analysis of the ComFa formulations, and review of the literature, suggested that the OFSP food matrix would promote iron absorption, and that OFSP ComFa would have more bioavailable iron compared with Weanimix [32], but this was not found in this study. Although OFSP ComFa contained 32-, 393-, and 7-times higher levels of ascorbic acid, β-carotene and fructose, respectively, than found in Weanimix, apparently these enhancers did not counteract the inhibitory effects of the approximately 1.8 times higher levels of OFSP ComFa polyphenols (Table 1). Ascorbic acid, β-carotene and fructose have been shown to improve the bioavailability of iron from foods [14,15,16], however, for ascorbic acid and fructose, this effect is dose dependent, affected by the food matrix, and diminished or eliminated by polyphenols [16,37,38]. Sweet potato has high concentrations of a range of different polyphenols [19,20], and although knowledge about the inhibitory effects of all the specific polyphenolic compounds in sweet potato is lacking, the results of this study suggest they are strong inhibitors of iron uptake. This is consistent with evidence demonstrating that polyphenols are potent inhibitors of inorganic iron absorption, and limit food iron bioavailability [21,22]. Indeed, the results of this study suggest that the presence of polyphenols is critical to the overall iron bioavailability of foods, even when large amounts of known iron absorption enhancers are present. One caveat to this is that there is evidence that the food matrix itself also impacts on the relative importance of inhibitors and enhancers. Two studies looking at legume-based foods found that low iron bioavailability was associated with higher phytate/iron molar ratios and total phytate but not the amount of polyphenols [39,40], in contrast with the findings of the current study.

It is noteworthy that the iron content (Table 1) in each of the tested formulations was similar, underscoring the fact that inorganic iron bioavailability is not primarily determined by absolute iron levels, but rather by the biochemical form of dietary iron, in combination with the balance of inhibitors and enhancers of iron absorption in the food matrix.

Interestingly, ferritin formation with OFSP ComFa was higher than with CFSP ComFa (Figure 1) despite similar levels of polyphenols and ascorbate, and *lower* TDF and phytate levels in CFSP ComFa (Table 1); this suggests that other factors accounted for the difference in iron bioavailability between these two sweet potato-based foods. Fructose and β-carotene levels were higher in OFSP ComFa compared with CFSP ComFa; therefore, whether these enhancers were responsible for the observed difference in ferritin levels between OFSP- and CFSP ComFa was investigated. Experiment II tested whether exogenous fortification of CFSP ComFa with fructose and β-carotene at the concentrations found in OFSP ComFa would result in equivalent iron uptake from the two different types of sweet potato-based foods. However, neither the addition of β-carotene alone, nor β-carotene and fructose together, improved the amount of bioavailable iron from CFSP- compared with OFSP ComFa (Figure 2). 

The negative findings of Experiment II may be the result of the specific single type of β-carotene used (versus the variety of forms naturally occurring in foods), or because, in order for β-carotene to improve iron bioavailability, it needs to be present together with iron in the original food matrix. Alternatively, in this food matrix β-carotene may not act as an iron uptake enhancer.

The mechanism by which fructose increases iron bioavailability is uncertain, although a recent study suggested that fructose reduces non-haem ferric iron to the more bioavailable divalent ferrous form [16]. However, the enhancing effects of fructose on iron bioavailability demonstrated in the previous study were negated by both phytates and polyphenols at levels lower than those found in either OFSP- or CFSP ComFa [16]. Thus, the negative findings in this current study are consistent with previous work, and indicate that higher fructose levels do not explain the different iron bioavailability of OFSP- compared with CFSP ComFa. In summary, the exact reason for the difference in iron uptake between the two sweet potato-based formulations is unclear.

It is known that sweet potato colour may indicate the presence of various polyphenolic compounds; research analysing sweet potato cultivars has demonstrated differences both in type, and amount, of polyphenols between cultivars with different colours [20]. The results of the present study suggest that different polyphenolic compounds present in cream-fleshed *versus* orange-fleshed sweet potato might account for their differing iron bioavailability. A recent study by Hart *et al.* using different coloured beans found that polyphenol type varied with bean colour, and specific polyphenols had differing effects on iron bioavailability at equivalent concentrations [41]; these findings are consistent with the hypothesis proposed above, that although the total polyphenol concentrations of CFSP- and OFSP ComFa were the same, differences in types of polyphenols led to differing iron uptake. A limitation of the current study is that only total polyphenol levels were measured; the findings of this research suggest a need for specific analysis of polyphenols found in sweet potato-based foods (and the sweet potato cultivars used to make them) for their effects on iron bioavailability.

The amount of iron available from CFSP ComFa was significantly lower (1.3 times) than that of Weanimix (Figure 1) although CFSP ComFa contained higher amounts of ascorbic acid (37-fold), β-carotene (37-fold) and fructose (3-fold) than Weanimix (Table 1). Furthermore, Weanimix had 5.5 times the amount of phytate compared with CFSP ComFa, and the iron:phytate molar ratio of CFSP ComFa was 1:0.92; this molar ratio would be expected to have minimal inhibitory effects on iron uptake [42,43]. TDF levels were higher in CFSP ComFa compared with Weanimix, which might, in part, explain the lower bioavailability. Perhaps more significantly, CFSP ComFa, similarly to OFSP ComFa, contained approximately twice the amount of total polyphenols compared with the cereal-based complementary foods (Table 1), which would be predicated to decrease iron absorption [21,22,41]. Significantly, the non-haem iron inhibitory effects of polyphenols are only partially counteracted by ascorbic acid at the levels found in the ComFa foods [44], as opposed to phytates for which ascorbic acid has been shown to more completely reverse the inhibitory effects at lower concentrations [43,44]. Two recent studies have also suggested that polyphenols decrease haem iron bioavailability [23,45]; therefore, uptake of the haem-iron in CFSP ComFa (from the added anchovy powder) might also be decreased. There are no data (to the authors’ knowledge) about the effects of β-carotene on polyphenol-related inhibition of iron absorption. In summary, the research conducted in this study indicates that the decreased iron uptake seen with CFSP ComFa compared with Weanimix is most likely secondary to inhibition by polyphenols.

Overall, the findings of this study support the well-documented inhibitory effects of polyphenols on iron bioavailability, and also that this inhibition is only partially reduced by iron absorption enhancers. OFSP ComFa had higher concentrations of phytate and TDF than CFSP ComFa, but more bioavailable iron, and polyphenol levels were almost identical. Thus, the better iron bioavailability of OFSP ComFa is not explained by differences in absolute polyphenol levels, suggesting that CFSP ComFa-specific phenolic compounds were the most potent inhibitors, or that there are other unidentified iron uptake inhibitors in cream-fleshed sweet potato. The results of this *in vitro* study, demonstrating low iron bioavailability from Weanimix enriched with fish powder, confirm earlier findings of a randomised, community-based feeding trial, which found that infants fed enriched Weanimix for six months had decreased plasma ferritin and haemoglobin levels at the end of the intervention [46].

Although the findings of this study did not confirm the earlier suggestion that sweet potato-based foods would be less inhibitory on iron bioavailability compared with cereal-based formulations [32], these foods could be a good dietary source of other essential nutrients such as ascorbic acid and provitamin A (β-carotene) when used as a home produced complementary food. Furthermore, whilst the Caco-2 cell model overall accurately predicts whether substances enhance or inhibit iron uptake, it may not always precisely predict the magnitude of the effect [47], therefore *in vivo* studies to confirm these results are warranted.

## 5. Conclusions

CFSP- or OFSP ComFa are not better sources of bioavailable iron compared with non-commercial cereal-based weaning foods as measured using the Caco-2 cell *in vitro* digestion model. However, they may be a good source of provitamin A. Furthermore, sample preparation may affect iron bioavailability, and thus it is possible that sweet potato-based complementary foods prepared in a different manner might have more bioavailable iron. Studies to identify and analyse polyphenols from different sweet potato cultivars, and their specific effects on iron absorption, and investigation of the interaction between sweet potato polyphenols, phytates and fibre in the presence of ascorbic acid, β-carotene and fructose, could help to identify optimal sweet potato cultivars, and sample preparation methods, to be used as a basis for complementary foods. Follow up *in vivo* studies to confirm results are also warranted.
